# Integrated Polymeric Sensors in Heart and Blood Vessel Monitoring: A Review

**DOI:** 10.3390/s25237178

**Published:** 2025-11-24

**Authors:** Vytautas Bučinskas, Jūratė Jolanta Petronienė, Gediminas Vaičiūnas, Nikolaj Šešok, Andrius Dzedzickis

**Affiliations:** 1 Department of Mechatronics, Robotics and Digital Manufacturing, Faculty of Mechanics, Vilnius Gediminas Technical University, Plytinės Str. 25, LT-10105 Vilnius, Lithuaniajurate-jolanta.petroniene@vilniustech.lt (J.J.P.);; 2 Department of Transport Technological Machines, Faculty of Transport, Vilnius Gediminas Technical University, Plytinės Str. 25, LT-10105 Vilnius, Lithuania

**Keywords:** cardiovascular system sensor, force sensor, sensor in stent, biodegradable sensor, implantable sensor, polymer, polydimethylsiloxane

## Abstract

This paper presents recent progress (2019–2025) in the role of polymer-based sensors implemented for heart and blood vessel monitoring. The existing variety of polymers, of synthetic and natural origin, allows the creation of sensors tailored to specific needs, to monitor heart health status for invasive cardiovascular surgery. Polymers, in combination with nanomaterials, nanostructures, or nanostructured materials, enhance the characteristics of force sensors. The review discusses implantable sensors applied in healthcare, especially for cardiovascular system monitoring, which provide the possibility to prevent the development of pathology or to control existing pathology. Additionally, the emerging need for biodegradable devices requires a review of the polymers already used. The quality and accuracy requirements of sensors for self-monitoring and health status control in medical institutions vary; yet needing a variety of sensors does not reduce the importance of finding sensors that are more accurate or more comfortable to wear. Sensors suitable for short-term use become important in the postoperative period, with the need for biodegradable polymers. This review focuses on publications that provide an analysis of the sensors as well as their potential for medical purposes. Our review focuses on polymers applied to force sensors for cardiovascular system monitoring. Overall, this review explores the paths of innovations in the field of novel technologies for self-monitoring of health. Finally, future research directions reported in the selected articles for cardiovascular care sensors are discussed.

## 1. Introduction

Cardiovascular disease (CVD) has become a significant health problem globally [1]. While cardiovascular diseases (CVDs) have become one of the leading causes of mortality worldwide over the last few decades [2,3], some sensors can help to monitor this health problem. The main pathology of cardiovascular diseases is atherosclerosis or the hardening of blood vessels, wherein the narrowing of the arteries disrupts the blood flow and regular supply of oxygen and nutrients, leading to heart attack and stroke [4]. The common treatment today, with the advancements of modern medical sciences, is stenting [5,6]. Cardiovascular stent coatings require much investigation, to support the multifunctional properties of stents [7]. Although most smart stent technologies do not have an integrated sensor, there is potential to integrate microsensors to monitor situations in real time for precise drug delivery or to adjust the therapeutic effects [8]. J. Park [9] reviewed the latest medical tools to incorporate sensors. The limitations of metallic stents have led to the development of polymer stents, which, in turn, opened up the field of biodegradable shape-memory nanocomposites for controlling heart diseases [10]. One possible way to control the disease is with implantable stents equipped with sensors. Polymer-based medical implantable devices require biocompatibility over the entire lifecycle. The capabilities of developing cardiovascular monitoring and healthcare devices are based on the unique properties of polymers [11]. Another decision involves the strut patterns of various stents, which can cause side effects from non-biodegradable metal [12]. Self-powered mechanical sensors can enable long-term real-time monitoring by cardiovascular devices to detect abnormalities, thus allowing early intervention [13]. The diversity of medical equipment is a huge achievement, allowing one to choose the most appropriate solution for a specific patient; however, the abundance of information can complicate knowledge management and information accessibility.

This review focuses on an analysis of the achievements in cardiovascular system stents containing biometric parameters’ polymer-based force sensors, incorporated into the stent structure, without delving into the polymer synthesis or physicochemical properties of polymer manufacturing and without considering additional functions such as drug delivery. On the other hand, we mention bioabsorbable smart stents and future perspectives that may evolve this area of research. This review focuses solely on the structural function of cardiovascular stents with integrated sensors, depending on the selected polymer. This research is presented to demonstrate the critical role of polymers in the development of stents with embedded sensors, using relevant examples from publications that illustrate a variety of solutions. The presented statistics on publications related to this topic may be useful for developers to assess which path is most promising for developing a new generation of stents with polymer-based sensors. As in most medical device development, polymers play a crucial role. Even well-known designs can be significantly improved with the use of new-generation polymers.

The main goal of this study is to inform the scientific community and physicians about the innovative equipment, polymer developers about which products have been successful, and sensor manufacturers about which materials provide better results, in order to produce a novel sensor. The topic of microelectronics is also discussed, where the essential interaction between molecules and nanostructures takes place.

For clarity, the aim can be expressed by these three objectives:

- To analyze the achievements in cardiovascular system stents containing biometric parameter polymer sensors;
- To demonstrate the critical role of polymers in the development of stents;
- To inform the scientific community, physicians, and polymer developers about the innovative equipment.

The remaining part of this paper is organized as follows: Section 2 presents the method of article selection, indicating the main ideas of the investigation; Section 3 presents the fundamentals of force sensors integrated in cardiovascular stents with polymeric structures, and Section 4 discusses polymer-based smart stents with integrated sensor technologies. The paper concludes with the results obtained and highlights the observed trends in the researched area.

## 2. Methods of Investigation

**Figure 1:**
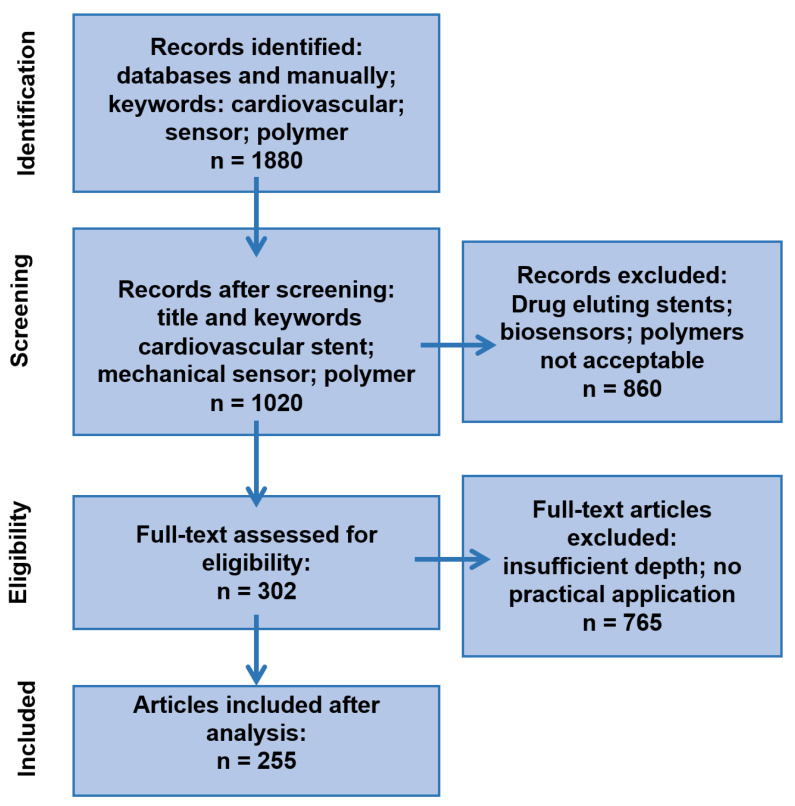
PRISMA-style diagram illustrating the number of articles at each stage of investigation/reviewing.

This research analyzes interdisciplinary data on the role and influence of polymers in the development of cardiac stents containing an integrated mechanical sensor, published between 2019 and October 2025. The selected data underwent a three-step procedure: sorting and searching by keywords, screening by title, and detailed reading, with a focus on polymer application in force sensors used for cardiovascular system monitoring. Statistical data are used to identify trends that may lead to promising future research. The statistical analysis was performed according to the PRISMA procedure [14]. The results of the screening are presented in Figure 1. After statistical analysis of the selected articles, the results were presented in the following topic groups:

- Polymers applied in force sensor manufacturing.
- Materials to improve the properties of polymer-based force sensors.
- Polymers’ role in “smart stent” elaboration.
- Polymer-based microsensors for cardiovascular monitoring.
- Biodegradable polymers used in force sensor manufacturing for “smart stents”.

**Figure 2:**
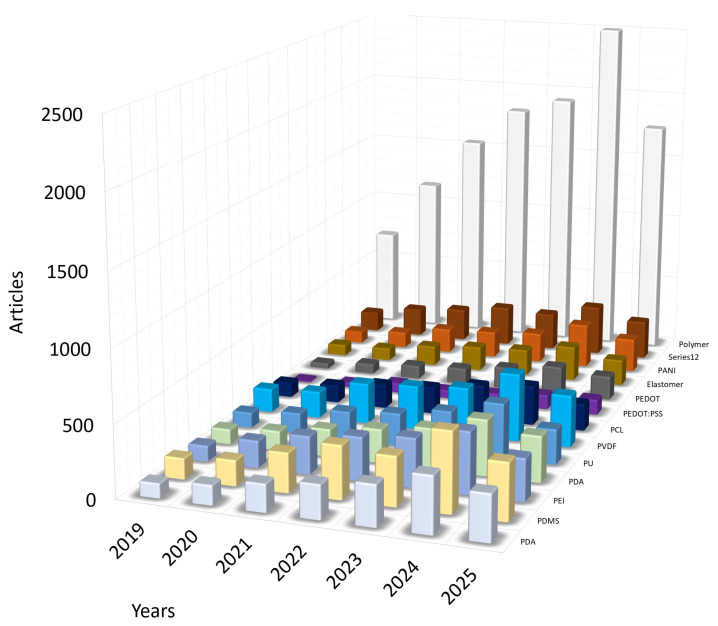
Statistics of published articles 2019–2025 on keywords. Visual representation of the distribution of articles by polymer names in articles on cardiovascular stent manufacturing.

The search was performed in several stages. For example, during the first search stage, articles with the mentioned keyword “polymer” were selected, and after this selection, the review was performed using the specific name of the polymer. An additional analysis was performed using the name of the particular polymer and including other keywords important for the review, so that the content corresponded to the selected topic: the polymer is used in the structure of the force sensor, and the sensor itself is integrated into the cardiovascular stent. For articles with the keywords ‘Polymer’ and ‘Cardiovascular sensor’, it is observed that the number of publications is increasing annually, and in 2024, it exceeded two and a half thousand. However, upon examining the specific name of the polymer, no dominant polymer was found, and the numbers of publications on the chosen polymer names are presented in Figure 2.

Luminescent molecularly imprinted polymers or other polymers with wide applications in biosensors are not discussed in this work. One of the aims of this work is to evaluate the evolution of “smart polymers”, those that can change their mechanical properties in response to an electric field and vice versa. The quality and suitability of polymers in sensor manufacturing are already well-established. The application of polymers in sensors for cardiology has been reviewed from several aspects. Polymers play a scaffold and frame role, and electrically conductive polymers become the main part of the sensor, converting mechanical energy into an electrical current. Biomedical polymers are either of natural origin or synthetic varieties, which are friendly to human health, biocompatible, and do not cause mutagenic, carcinogenic, or immunogenic reactions. Natural biomedical polymers, such as polysaccharides, are promising in the manufacturing of biodegradable devices. Synthetic biopolymers have more stable performance and are easier to produce. The discussion of biomedical polymers is intended to serve as a source to develop innovative polymers and apply known research in clinical practice. In this research, Google Scholar, ScienceDirect, IEEE Xplore, and PubMed were utilized as research engines. The analyzed publications were selected from the ScienceDirect, Springer, Google Scholar, IEEE Xplore, Research Gate, Web of Sciences, MDPI, and Wiley databases using the following keywords: electroactive polymer, conductive polymer, piezoelectric polymer, biodegradable polymers, carbon nanomaterial, cardiovascular, conducting polymers, sensor, force sensor, Kapton, MEMS, microsensors, nanostructures, natural polymers, polymer, Polydimethylsiloxane (PDMS), Polyaniline (PANI), Poly(acryl amide) (PAAm), polylactic acid (PLA), polydopamine, polypyrrole (Ppy), poly(ether-*b*-amide), polyimide (PI), poly(ɛ-caprolactone), poly(vinyl alcohol) (PVA), poly(vinylidene fluoride) (PVDF), Polyvinyl chloride (PVC), Silk fibroin, thermoplastic polyurethane (PU), strain sensor, sensor in stent, smart stent, TENG, B-TENG. Some of the numbers of references are shown in tables to demonstrate the novel decisions and trends in sensor manufacturing for cardiovascular system monitoring. The statistical data were extracted from the ScienceDirect database. It is evident that the specifics of the chosen topic currently limit the use of certain polymer classes in the production of implantable cardiovascular stent parameter sensors; however, the situation may change in the future, as new solutions emerge.

## 3. Principles and Fundamentals

Medical devices are facing increasingly diverse performance requirements. Y. Wu [15] presented new bioresorbable mechanical sensors for vascular stents and the fundamental design principles of mechanical sensors.

Printed electronic vascular stents with integrated sensors can monitor pressure, blood flow, and arterial stiffness [16]. Although the variety of modern stents produced for cardiovascular system surgery is in the hundreds, their functionality is determined by the sensors built into them and the polymers employed [17].

Conductive polymers demonstrate the ability to conduct electrons through their conjugated backbone [18,19,20]. In some cases, molecular imprinted polymers have been applied for stent surface modification, making the stent a multifunctional device [21,22,23]. The application of conjugated polymers in stents has opened up drug-delivery functions [24]. Shape memory polymers are a promising material for stent manufacturing [25]. Personalized stents with drug-eluting properties and bioresorbable stents have received attention due to novel polymeric materials [26]. Shape memory polymers have a role in stent manufacturing [27]. Shape memory polymers (SMPs) utilizing magnetic fields represent another family of smart materials applicable for medical device manufacturing, where SMPs utilize thermal energy to activate their shape memory [28,29,30]. The thermosetting polymers have had successful applications in a variety of stents [31,32,33,34,35,36]. To avoid the complications of in-stent restenosis, inflammations caused by metal structures, stents have experienced various forms of improvement, including smart sensor-based devices [37].

Modern sensors allow implantable medical devices to perform an increasing number of monitoring functions. The sensors integrated into stents enable the detection of blood clots by impedance monitoring [38]. The elaboration of multifunctional stents with integrated sensors and biosensors has become an interdisciplinary research area requiring collaboration between medical doctors and engineers, adapting the latest sensor technologies for real clinical applications [39]. With the rapid development of flexible and stretchable electronics, the mechanical compatibility and biocompatibility of electronic devices have been significantly improved, and a series of new conductive polymer materials or semiconductors with excellent stretchability have been adapted [40,41,42,43].

Developers of modern sensors take into account not only the standard requirements for flexible wearable or implantable sensors but also features such as the product’s antibacterial function, electromagnetic shielding, waterproofing, and self-healing [44,45]. The variety of synthetic and natural polymers is currently quite large, but the suitability of these polymers for implantable wireless, self-charging, or biodegradable devices still requires better solutions [46,47,48,49,50]. The manufacturing of polymers for medical purposes is a complex and responsible field of science, as the final product must meet extremely high standards [51,52]. Electroactive polymers exhibit shape changes in an electrical field and are useful for tissue regeneration. The application possibilities are expanded when used in conjunction with pressure, strain, or tactile sensors [53]. Polymers for implantable devices have the requirement of biocompatibility and biodegradability in some cases, with appropriate biochemical reactions to surrounding tissues, and useful physical properties [54].

Conductive hydrogels with ionic–electronic conduction have application in tissue regeneration, biomedical applications, and stents [55]. Conductive polymers have a tremendous impact on implantable devices [8,56]. Biodegradable polymeric stents are rightly called a new step in cardiovascular disease monitoring and treatment [57,58,59]. The collection of smart and intelligent materials for cardiovascular healthcare is rapidly growing [60]. With the application of stents as a treatment, there is a need for accessible and cost-effective methods for restenosis detection or smart stents with microelectromechanical systems (MEMS) [61]. Recently identified as a new technology, they are already being applied in medical equipment. R. Herbert presented the aerosol jet printing system [62], where a ‘smart’ stent can measure the pulse rate and blood pressure. Although many stent implantation operations are performed every year, the most common complication of restenosis still poses a significant risk to patients [63,64]. Recently, wireless communication for medical implants has become crucial to real-time health monitoring. However, existing electromagnetic wave-based methods of communication face some limitations due to their specific signal attenuation, and ultrasound methods require specific integrated circuits or special metamaterial-based sensors [65]. Electromagnetic-based communication methods have high attenuation and limited penetration into biological tissues, but ultrasound connections can address these shortcomings, typically requiring highly customized and complex designs of implant electronics or physical changes to the implanted metamaterials [65]. Differences in the area of the inferior vena cava (IVC) and folding are early markers of overload and predict the risk of heart failure (HF) [66]. Despite the availability of advanced medical solutions for treating cardiovascular diseases, prevention programs remain equally relevant. Hence, continuous and efficient monitoring of the cardiovascular system using pressure sensors becomes essential for the prevention of heart failure. Constant monitoring of the vascular system by devices attached to the human skin has attracted attention to determine the early stage of cardiovascular diseases to prevent mortality [67,68,69,70,71]. A single-device platform for cardiac diseases is desirable [72,73]. Mechanical energy converting to electrical energy, or triboelectric sensors, can be applied for blood pressure monitoring [74,75,76]. Recently, highly elastic fiber sensors have attracted great interest due to their applications in wearable electronics, human–machine interfaces, and implantable biomedical devices [77]. With advanced material processing techniques, the new generation of physical sensor manufacturing has begun.

The quality of sensors is improving significantly with the use of carbon materials and nanostructures in their production [78]. Materials, such as metals, metal films, oxides in nanostructured form, nanostructured polymer surfaces, hydrogels, and foam-like structures, have opened a new era in the manufacturing of health monitoring devices [79,80,81,82].

Another essential issue in wearable sensor system manufacturing is the biodegradability of these products and the use of human-friendly technologies in all steps of manufacturing [83,84,85,86]. For example, some nanomaterial production processes result in toxic byproducts, which are unacceptable due to their negative environmental impact, as they release toxic gases or chemicals in other physical states [87,88]. The processing, storage, and transportation of toxic waste increase the cost of the final product and are unattractive to the workers producing it, as well as to organizations concerned with environmental protection, and to consumers who are ultimately responsible for its consumption. With advanced material processing techniques, a new generation of physical sensor manufacturing has begun. The quality of sensors is improving significantly with the use of carbon materials and nanostructures in their production. Patterned structures of polymers give the surface exceptional biological, adhesive, wettable, and optoelectronic properties, which are applicable for sensing applications [89,90]. Ideas of sustainability and environmental protection also drive the search for new solutions. When developing implantable medical devices [91], the scientists and engineers prioritize wireless connectivity, biocompatibility, and structural comfort. Hence, a power supply becomes essential to provide longevity in the human body. Some of the latest data on several different types of sensors and sensing systems integrated into endovascular catheters are presented by C. Kaminski [92]. The magnetic sensors, which are independent of light transparency, interact better with the skin surface and maintain accuracy during movement, making them suitable for future health status monitoring devices [93]. Optical and magnetic sensors are critical to advancing heart health monitoring. Their integration will improve our ability to monitor, diagnose, and manage cardiovascular health, promising a more proactive and patient-centered approach to care [93].

Biodegradable energy sources have their own benefits in contrast to retractable non-biodegradable ones, whose removal can cause health risks [94]. For example, B-TENG made from PLA in a health monitoring device can ensure healthcare and bio-resorb at the scheduled time [95]. Biodegradable materials from renewable sources and synthetic materials can be hydrolyzed or can be modified with additives like antioxidants [96]. Since both temperature and pulse signal monitoring are complementary diagnostic tools for cardiovascular diseases, the incidence of certain coronary diseases, such as hypertension, tachycardia, and bradycardia, can be predicted using strain sensors [97,98,99,100,101,102]. Hence, the increase in pulse rate shows a strong positive correlation with the prevalence of coronary heart disease, where tachycardia denotes a pulse rate exceeding 100, while bradycardia is defined as a pulse rate of 60 beats per minute [103]. Thus, pulse rate measurement has become an indispensable diagnostic procedure for patients, just as force sensors have become essential. Self-powered wearable devices for health monitoring have made significant progress in the last few years [104,105]. The operating mechanisms of self-powered sensors are piezoelectric, electrostatic, and electromagnetic [106,107]. Biodegradable devices, especially triboelectric nanogenerators (B-TENGS), have emerged as an innovative technology in the field of self-powered implantable devices field [108,109,110]. Biodegradable nano-sensors are critical devices for the detection of postoperative complications [111,112,113,114,115]. Biomedical polymers can be identified as key materials at the forefront of medical advances, offering innovative solutions for disease prevention, diagnosis, treatment, and clinical use due to their exceptional physical and chemical properties. Innovations in healthcare are constantly discussed in reviews [116]. However, the abundance of information is so great that it is necessary to frequently systematize the new messages that appear. The latest trend in cardiovascular system monitoring is heart health apps, which are accessible on the Apple App Store and Google Play stores or other sources locally [117]. This situation entitles a separate area of health self-monitoring where sensors are fully integrated into consumer products. A machine learning approach with non-invasive sensors was presented by P. Arpaia [118]. Machine learning is promising for the future, with two key benefits: it will enable the real-time investigation of patients’ cardiovascular risk and provide a database for future research. There is a vast supply of devices and health monitoring sensors, suitable for specific operating conditions [119,120].

## 4. Polymers Applied in Force Sensor Applications

**Figure 3:**
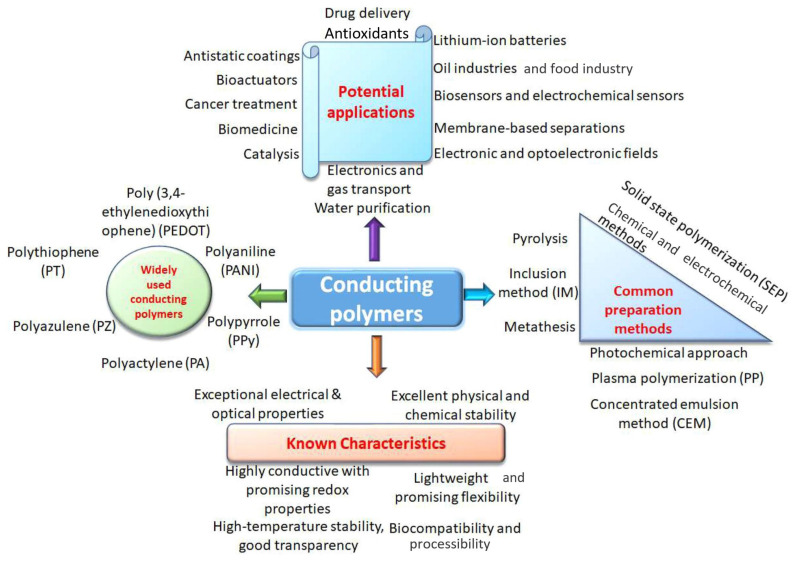
Known characteristics, potential applications, and common synthesis methods for CPs production [124].

The specific structure of natural tissues necessitates the development of an artificial system that functions similarly to well-adapted natural tissues, enabling effective force sensors to record the physiological properties of the human body. Thin film device manufacturing arises with emerging carbon nano-materials and conductive polymers [121]. Conductive polymers can be synthesized directly or be produced, for example, by doping or some chemical treatment: electrochemical polymerization or potentiodynamic method [122,123]. The basic information about conductive polymers is presented in Figure 3 [124].

The low detection limit and high sensitivity issues require the employment of conductive polymer composites with nanomaterials or nanostructured [125] and wrinkled surfaces [90,126,127,128,129]. Flexible substrates that replace rigid materials enable sensors to optimize their best features for the requirements of wearable devices. Polymer films are the desired material for developers of human body parameter monitoring systems. Conducting polymers, with their unique material properties due to their electrical, optical, and biocompatible properties, are becoming a promising material for medical purposes. Z. Zhou [125] proposed PANI nanowires using interfacial polymerization for wrinkled microstructure through repetitive stretching for a pressure sensor to track dynamic and static activities. The polymer sensor surface structure is essential for sensor functioning [130].

### 4.1. Materials to Improve Polymer-Based Force Sensors Properties

Modern sensors are typically made of polymers enriched with nanostructures or microstructures. Nanomaterials play a crucial role in the applications of the new generation of wearable electronics [131,132,133], but nanomaterial-based actuators are still in the early stages of successful development [134]. To produce micrometric-size sensors, it is essential to understand sensor physics and processes at the molecular level. The synthesis of nanomaterials with predefined structures, such as 0D [135], 1D [136], 2D [137], or 3D [138], has shown promising results in the development of these sensors [139]. Developers often point out that, despite the sensor’s functionality, the existing methods have shortcomings that prevent the production of high-quality and defect-free sensing layers [140,141,142]. To resolve this issue, the development of suitable 2D materials with appropriate features, low cost, and a simple method for mass production is an area of focus for future material scientists [143]. The application of nanoparticles [144,145,146] in sensor manufacturing has led to the development of novel technologies, such as pharmaceutical nanotechnology, enabling multifunctional implanted devices, biocompatible [147] and biodegradable devices [148], as well as antimicrobial nanocoatings [149] and hydrogels [150].

The microstructure-frame-supported organic thermoelectric (MFSOTE) material-based devices are promising for health monitoring. MFSOTE materials are, for example, poly(3,4-ethylenedioxythiophene), poly-(styrenesulfonate), (PEDOT: PSS), and PU [151]. Recently, flexible electronic devices have attracted significant interest as an alternative to traditional rigid metallic conductors for personal healthcare, as traditional conductors have weak properties: poor stretchability, sensitivity, and strength, as well as low conductivity and a single sensing function. The application of metals, ceramics, and other solid materials in sensor manufacturing was renewed with the discovery of the significance of cracks, micropores, and rough surfaces in the development of such sensors [152].

### 4.2. Polymer Role in “Smart Stent” Elaboration

As stated by many authors on the cardiovascular stent topic, cardiovascular stent angioplasty is the best option for treating coronary artery diseases. Despite successful developments in this field, when drug-eluting stents, short-term stents, and bioabsorbable stents are elaborated and introduced into cardiovascular healthcare routine, several clinical complications related to stent applications, such as thrombosis and restenosis, have occurred [153]. Thus, it is first necessary to identify what and how adverse effects occur when using stents in the cardiovascular system, as well as the challenges for developers. The corresponding “smart stent” systems with sensors are receiving research interest, given their potential to mitigate/reduce several undesirable stent-related consequences [153]. Smart stents focus on integrated pressure sensors [154].

The technical evaluation of stents can be performed using optical polymers and technologies such as optical coherence tomography (OCT), which allows obtaining high-resolution cross-sectional images of the coronary arteries, allowing the evaluation of the results of stent implantation [155]; however, polymers with optical properties are not the subject of implantable sensors in stents.

**Figure 4:**
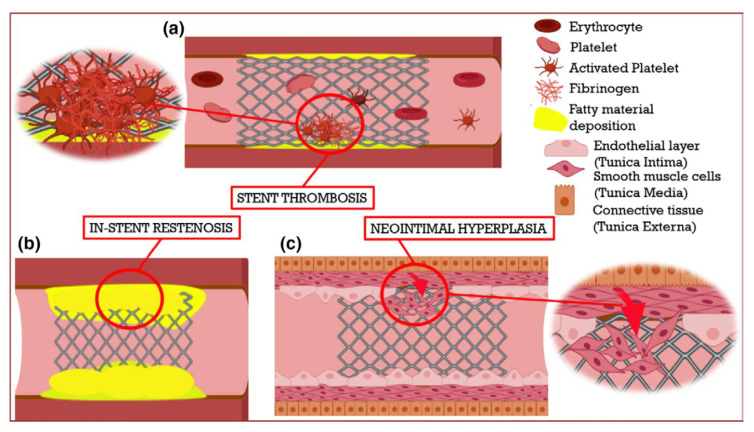
Schematic representation of the significant limitations associated with cardiovascular stents [153]. (**a**) Stent thrombosis occurs due to the formation of a blood clot inside the stent region. (**b**) In-stent restenosis develops with the re-occurrence of fatty deposition (as shown in yellow) in the stenotic region. (**c**) After a stent is implanted, delayed endothelialization or endothelial denudation can lead to the migration of smooth muscle cells from the middle layer of the blood vessel, resulting in smooth muscle cell proliferation and neointimal hyperplasia.

Figure 4 schematically represents stent-related complications. Y.X. Ang [156] presented restenosis treatment stages. Therefore, it is of extraordinary importance in the development of technologies to reduce the health risk and improve the clinical efficiency in cardiovascular surgery.

**Figure 5:**
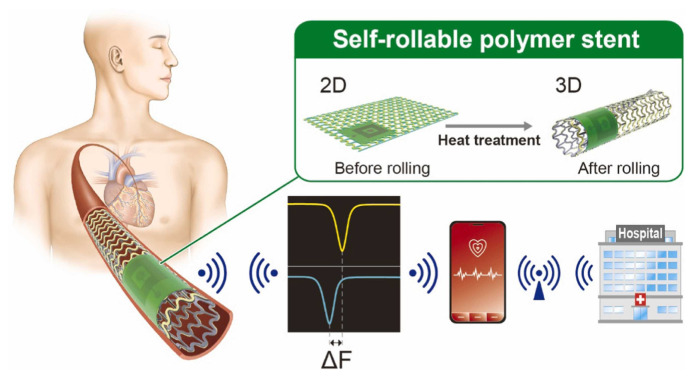
Schematic representation of a self-rollable polymer stent integrated with an LC pressure sensor [157].

A silicon-based self-rollable polymer stent with integrated pressure sensor (Figure 5) was presented by N.E. Oyunbaatar [157], using the MEMS technique to monitor blood pressure inside the arteries.

A serpentine-shaped wireless biodegradable polymer-based self-reported stent structure was presented by N.E. Oyunbaatar [158], which is capable of monitoring vascular abnormalities due to the incorporated capacitive pressure sensor. The authors reported successful stent implantation into the arteries of a three-dimensional phantom system, operating in various environments, including in vivo conditions. The sensor demonstrated a pressure sensitivity of 0.15 mm/Hg.

**Figure 6:**
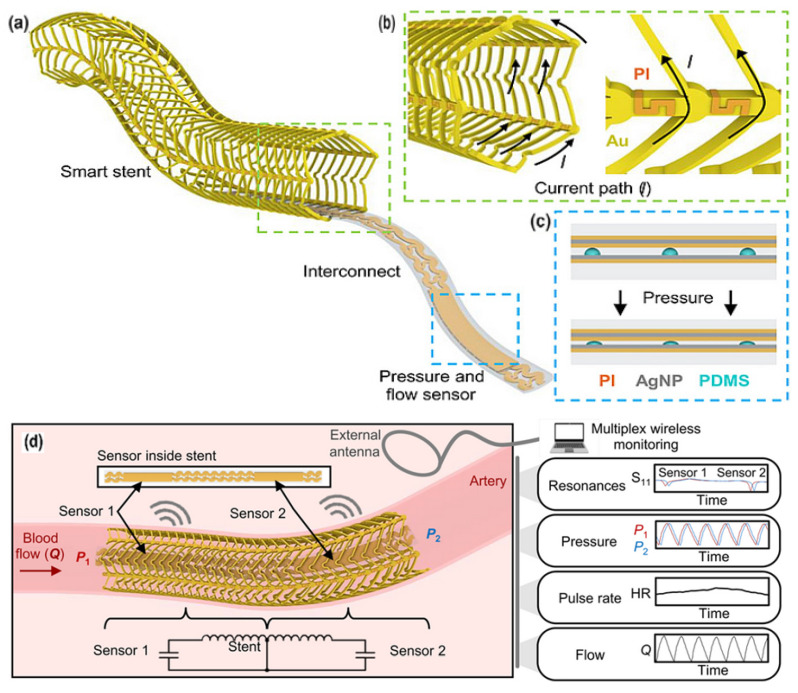
Fully implantable wireless vascular electronic system. (**a**) Illustration of the implantable electronic components; (**b**) inductive stent design using conductive Au loops and nonconductive PI connectors to achieve a current path resembling a solenoid (**left**) and a scanning electron microscopy (SEM) image of the stent (**right**). (**c**) Layers of the soft pressure sensor using a printed dielectric layer (**left**) and a photo of an index finger holding a simultaneous flow and pressure sensor (**right**). AgNP, silver nanoparticle; PDMS. (**d**) Illustration of the wireless design and sensing scheme to simultaneously monitor pressure, heart rate (HR), and flow [62].

The PDMS is valuable due to its biocompatibility and elasticity. PDMS has a low elastic modulus and high deformability, enabling dynamic surface compliance that makes it ideal for biological sensor devices [159]. The most common application of PDMS is to cover the functional layer of the sensor, thereby obtaining a flexible strain sensor. P. Sharma [160] proposed graphene nanoplatelets (GNP) and PDMS to fabricate a strain sensor that is widely applicable, from health status monitoring to monitoring of climate change effects. The construction of conductive polymers using PDMS has resulted in effective sensing functions, as supported by numerous examples. A simple shark skin-inspired pulse sensor made from PEDPT: PSS with PDMS was proposed by H.-H. Jang [161]. The PDMS-MXene, MoS_2_-based on a silicon substrate, for heart rate and UV detection, a self-powered and integrated triboelectric nanogenerator (TENG) was presented by A. Mirsepah [162]. S. Chen [163] developed a self-powered pressure sensor implemented for hierarchical elastomer, based on electrostatic nanogenerators designed for real-time blood pressure measurements. H. Fang [164] proposed a Real-time Health Evaluation System (RHES) using arch piezoelectric sensors for capturing pulse signals, produced using poly(vinylidene fluoride)–polydimethylsiloxane (PVDF–PDMS). A porous PDMS was applied in a pulse-wave hair cell structured sensor by M. Seok [165], which contains a 100/10 nm thick Au/Cr layer and PI. A flexible capacitive pressure sensor BaTiO3-doped PVDF electrospun fiber with PDMS microcylindrical structures elaborated as a dielectric layer was proposed by C.-R. Yang [166], with a sensitivity of 5 kPa^−1^. For non-conductive polymers, their conductivity can be improved by incorporating specific functional groups. The most popular conductive polymers are polyacetylene, Ppy, poly(p-phenylene), polythiophene, and PANI, all of which possess delocalized electrons in their π orbitals, facilitating electrical conduction [167]. In Figure 6, a 3D-printed patterned PDMS capacitive sensor integrated into a stent [62] is depicted, which successfully recorded data for 2 months and continued to function for an additional 3 months after implantation. The skin-conformable polymer/oxide p-type semiconductor phototransistor, used as a photoplethysmogram sensor for cardiovascular monitoring, was presented in the work by B.H. Kang [168].

PDMS is known for its high dielectric constant value, flexibility, and as a biocompatible polymer with wide application [169,170]. Some successful solutions were obtained by combining several polymers. Illustrations of the successful combinations of one selected polymer, PDMS, with other polymers in sensor manufacturing are presented in Table 1.

**Figure 7:**
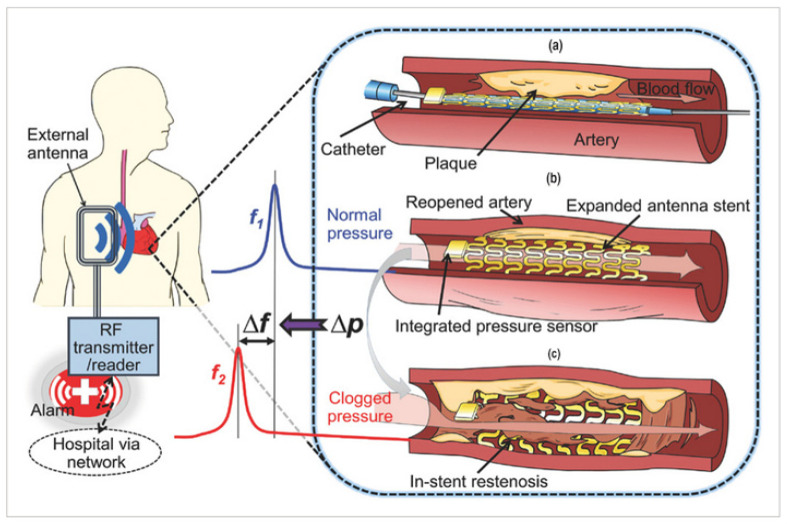
Conceptual schematic of the smart stent and its functions [189]: (**a**) a pressure-microsensor-integrated wireless stent crimped on the balloon catheter is positioned at the targeted stenosis site in the artery; (**b**) the smart stent is deployed by balloon inflation to start self-diagnosing while scaffolding the narrowed artery after removal of the catheter; the stent’s resonant frequency is at its nominal level (*f*_1_); (**c**) in-stent restenosis changes local blood pressure and shifts the stent’s frequency (to *f*_2_) as a sign of the problem; the implant is continuously monitored through a handheld wireless reader (GORE-TEX Stretch Vascular Graft, W. L. Gore & Associates, Inc., Flagstaff, AZ, USA) that sends out a warning of restenosis upon occurrence.

The fractional flow reserve (FFR) guidewire has a diameter of less than 200 μm due to its need to enter constricted blood vessels; the MEMS was elaborated and presented in K.-J. Moon [188], as shown in Figure 7. The sensor must transmit the collected data from its location. The sensor should be electrically connected, waterproof, and coated to withstand the pressure inside the blood vessel [188,189].

U.-V. Romero [190] introduced the nitinol and Ni-Ti alloy-based stent sensor, which has been successfully used for medical purposes, due to its pseudo-elasticity and strong corrosion resistance. Additionally, it features the ability to measure several physiological parameters, utilizing PVDF and PDMS. The nitinol-based health monitoring sensor (NHMS) contains an integrated TENG and has the ability to charge a commercial capacitor, enabling it to function as a self-powered sensor. It was developed to measure the heart rate, blood pressure, and breathing patterns. The PVDF is highly favorable due to its high residual polarization and excellent thermal stability, making it a good option for smart stent manufacturing. The PVDF presents as five different crystalline polymorphs: α, β, γ, δ, and ε phases [191], where β-phase PVDF generates the best piezoelectric properties [192]. The most negatively charged and, therefore, the most preferred material for triboelectric device manufacturing is polymer polytetrafluoroethylene (PTFE) [193]. L. Wang [37,194] proposed a hybrid Co/Cr-Poly(ɛ-caprolactone) (PCL)–Co/Cr-based stent with an LC wireless pressure sensor, which can provide an intravascular signal. The biodegradability of a PCL and PLA polymer stent with a wireless MEMS-based LC-type sensor was presented by J.L. Wei [195]. PAA/Fe hydrogel demonstrates an accuracy and sensitivity of 12 nF/kPa for pressure detection [196]. PU is an excellent and widely used polymer with good biocompatibility and mechanical properties, making it especially useful in orthopedic, skin sensor manufacturing, and cardiovascular system monitoring devices [197]. PPy is a conducting polymer that, when blended with natural directing agents, can be suitable for improving sensor performance [198]. Conductive polymer (CP) hydrogels are desirable materials in sensor manufacturing; however, hydrophilic CPs and hydrophobic CP networks often exhibit inadequate bonding in matrices, resulting in the loss of desirable mechanical and electrical properties required for sensor applications. Z. Sun [199] investigated the mechanism of supramolecular interactions and stated that the impact of external conditions for many conducting polymer hydrogels remained largely unexplored. Hydrogels are 3D polymer networks, a promising material in sensor manufacturing [200]. Hence, scenarios for the successful application of supramolecular conductive polymer hydrogels are a challenge for the future [199]. The application of nanotechnologies and nanomaterials in cardiology is a promising frontier in patient care [201]. With the development of novel nanomaterials and nanostructures, combined with novel tissue technologies, clinicians have gained tools for innovative methods of diagnostics, treatment, and prevention of cardiovascular diseases [201]. PANI nanofibers with a high thermal expansion coefficient exhibit a short-term response to temperature changes, as the charge carrier hopping rate between fibers depends on the temperature [202]. Hence, the special role of fibers in the charge transfer pathway is a critical aspect in sensor development [203]. The use of bioresorbable stents with SMPs and additive manufacturing is changing the healthcare sector [204]. The application of dry electrodes for long-term ECG monitoring is a promising technology [205], as wet electrodes for long-term health monitoring may cause skin disorders. The application of polymer-based sensors with nanostructures or nanomaterials is presented in Table 2.

As presented in the table above, most publications dedicated to pressure and temperature sensors can be divided into two categories: one focuses on the manufacturer creating and testing the sensor for medical suitability, while the other focuses on a careful investigation of the technical parameters of the developed product. Thus, the development of the modern sensor is not solely in the hands of specialists with knowledge of nanotechnologies or polymers. To improve medical sensors more effectively, collaboration between interdisciplinary laboratories and the availability of simulation and modeling equipment is necessary. Hence, every well-designed sensor can be included in medical suitability studies; however, there is a delay, due to the lack of medical assessment. Due to the unique properties of natural-origin biopolymers, such as starch in combination with other polymers, a new era of sensor manufacturing has been opened. Starch-based gels and soft starch-based composites have been applied in resistive, capacitive, piezoelectric, and triboelectric sensors to measure temperature, humidity, fluids, and enzymes, to detect a wide range of stimuli, and to monitor human body movements and physiological signals [232,233]. A natural polymer reinforced with suitable compounds in a nanocomposite opens up promising multidisciplinary applications in sensor manufacturing. The gelatin and oxidized sodium *O*-(carboxymethyl) cellulose hydrogel demonstrates good mechanical strength [234]. Methylcellulose was used as a stabilizer in J. Gao’s [235] investigation of stretchable sensors.

### 4.3. Biodegradable Polymer Used in Force Sensors for “Smart Stent”

Biodegradable materials enable customized medical devices that improve personalized medicine, which are increasingly produced from environmentally friendly polymers, plant-based, such as polysaccharides [236], alginate [237], cellulose [238,239], and starch [240], and protein-based [241], derived from animal tissues, such as collagen [242], chitosan, fibrin, silk, and gelatin [243]. Prototyping biodegradable compositions for stents, E. Hasanpur [244] investigated the composition of PLA/Mg and noticed that Mg particles act as nucleation sites and support the hydrolysis-induced degradation of PLA. Due to its good biocompatibility and relatively large piezoelectric coefficients, as well as its non-pyroelectric properties, PLA has applications in various sensors and energy harvesting devices. A PLA film-based stethoscope and pulse sensor was presented by J. Tian [245]. A zero-power consumption and implantable bias-free cardiac monitoring capsule working on the triboelectric effect was proposed by X. Qu [219]. It is plant-based, 3D-printed, and incorporates near-field communication technology, featuring an integrated acquisition/transmission module that meets many modern requirements for this type of equipment. Biodegradable and bioresorbable devices eliminate the need for the surgical removal of implantable devices, reducing the risk and associated healthcare costs [246]. The sensors, made from natural-origin polymers, can naturally decompose within the body, making them suitable for creating temporary and less intrusive medical sensors. Developed pressure sensors, biosensors, and chemical sensors based on biodegradable PLA with biodegradable magnesium metallic electrodes are presented in R. Omar’s [84] article. Electrospinning, using natural proteins, such as collagen, elastin, gelatine, or synthetic polymers, such as PCL, is applied in tissue engineering [247]. The electrospun nanofibers proposed by B. Oh [248] were used for tissue engineering. Polymers implemented in biological parameter measuring sensors are presented in Table 3.

High-density lipoprotein (HDL) natural nanoparticles are 10 nm in size [259]. M. Zahran [260] conducted a study on the suitability of natural biopolymers, such as carbohydrate polymers for the production of sensors: alginate, cellulose, chitosan, dextran, gelatin, gum, pectin, and starch and their compatibility with nanostructures of different metal oxides: Ag, Au, Cu, Fe, Ti, Bi, Co, presenting careful systematization. A functional flexible sensor alginate–gelatin sponge was introduced for human motion monitoring by Y. Fu [255]. It can be used in multi-field sensing systems and human–machine interface systems. Table 4 presents examples of natural polymers used in conjunction with synthetic ones in healthcare sensors.

Additionally, as a proof-of-concept, the sensor was integrated into a wound dressing and functioned as a smart wound dressing. The hydrogen-bonding network based on carboxyl styrene butadiene rubber and hydrophilic sericin non-covalent carbon nanotubes was employed as a multifunctional sensor to obtain weak and large deformations with a detection limit of 1% of strain and high sensitivity to thermal change in 0.01 °C; it was proposed for an intelligent health tracking system by M. Lin [180]. Self-healing hydrogels for bioelectronic devices have enabled the realization of the tissue-like mechanical device era, due to their biocompatibility and good adhesiveness. Despite some of the hydrogel’s poor parameters, sensors based on hydrogels are very popular. The importance of sensors and actuators in wearable and portable electronics is growing, driven by new applications and innovative solutions for equipment. Proposing a well-working sensor is essential to avoid the influences of environmental temperature, humidity, and light on sensor operation. Problems cannot always be solved by encapsulation or be compensated for by additional temperature sensors, feedback circuits, or other improvements [266]. Hydrogels from natural-origin materials demonstrate good adhesion, biodegradability, and conductivity. Silk fibroin exhibits properties beneficial in postoperative surgery, particularly when it is crucial to minimize secondary damage [264]. Cardio tissue engineering, combined with biodegradable sensors for short-term monitoring, has great potential in regenerative medicine. Silk fibroin, in conjunction with graphene oxide (GO), plays an important role, as this composition can form flexible carbon monoliths [267].

### 4.4. The Polymer-Based Microsensors for Cardiovascular Monitoring

**Figure 8:**
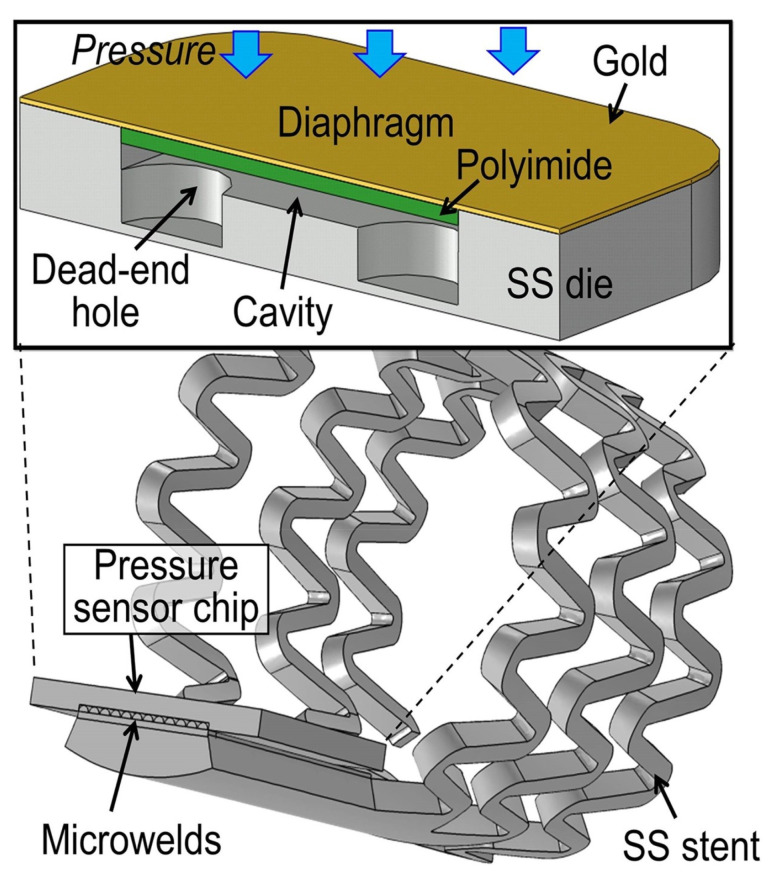
The cross-sectional structure of the developed capacitive pressure sensor chip and its micro-welding integration are illustrated with an example of the smart stent [272].

Microsensors play a key role in converting mechanical signals into electrical signals. In recent decades, significant progress has been made in sensing modalities, including temperature, pressure, inertial forces, magnetic fields, radiation, and other measurements, which are realized through various sensing modalities. Micromechanical systems (MEMS) are a group of devices, whose length is less than 1 mm [268]. The first successful single-crystal-based MEMS was introduced in 1954 by Smith [268]. The typical MEMS consists of a power supply, a signal transduction and processing unit, a micro-actuating element, a sensor, and an output action element [269]. The CardioMEMS™ HF system micromechanical sensor (Champion, CardioMEMS, Atlanta, GA, USA) is designed to assess pulmonary artery pressure [270]. N.-E. Oyubbaatar [157] presented a self-rollable polymer (SU-8) stent integrated with a passive inductor–capacitor resonator (LC) pressure sensor. The studies of other scientific groups were focused on the integration of sensors with stents using different techniques, such as laser welding, gluing, and bonding [271,272]. A stainless-steel (SS) chip of a capacitive pressure sensor (Figure 8) integrated by a micro-welding process for MEMS, proposed by X. Chen [272], has been found to offer both mechanical and electrical advantages over conventional conductive epoxy bonding. Fabricated sensors exhibit an average sensitivity of 100 ppm/mmHg over a gauge pressure of 250 mmHg.

The capacitive transducers used as pressure sensors in MEMS have become popular, especially in stents. X. Chen [189,273] presented a smart stent with a micro-sensor and a wireless interface to monitor restenosis through an implanted stent, working as a radio frequency wireless pressure transducer for tracking local hemodynamic changes upon narrowing conditions and expected to be used in clinical practice.

**Figure 9:**
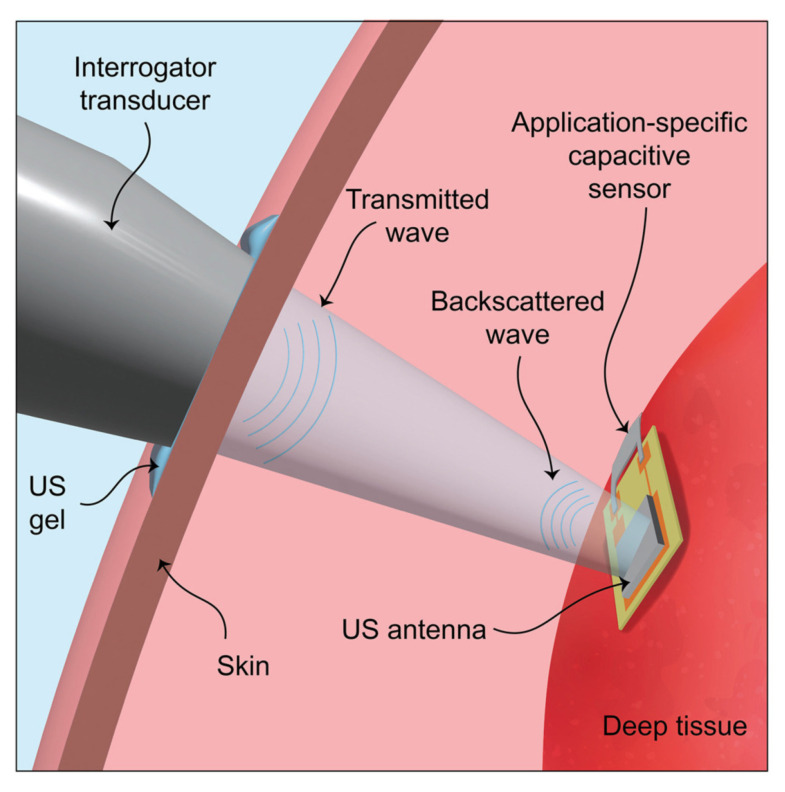
Schematic of the passive ultrasonic communication method. The ultrasonic antenna is placed on deep tissue, and the wireless communication link is established between the device and the external interrogator transducer [65].

The principle of operation of a capacitive sensor in deep soft tissues, elaborated by U.-C. Yener [65], is presented in Figure 9, where a piezoceramic US antenna connected to a capacitive sensor was used.

An inferior vena cava (IVC) implantable sensor with ultrasound data collection principle, operating controlled by computed tomography, providing daily self-assessment by patients was presented by P.R. Karla [66]. The FIRE1 system-type crown-shaped implant, incorporated in a belt antenna, deployed in the IVC between the hepatic and renal veins, was presented by W.S. Sheridan [274]. Triboelectric sensors demonstrate a fast response time, high sensitivity, and versatility, which can help to improve the functionality of cardiovascular monitoring devices [275]. Triboelectric nanogenerators (TENGs) have been considered an effective method for self-powered systems for a decade.

**Figure 10:**
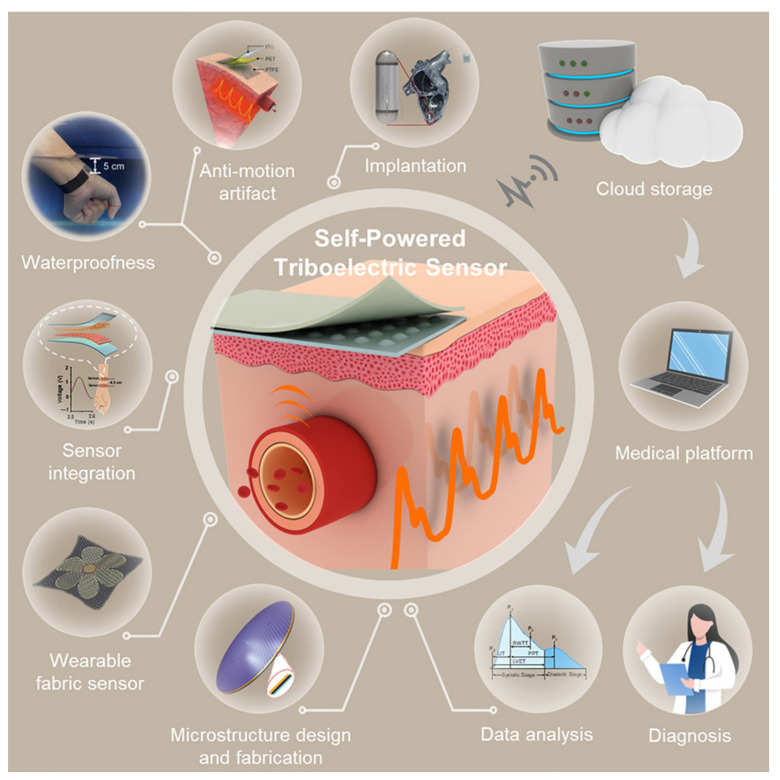
Self-powered triboelectric sensors [276].

Triboelectric sensors (Figure 10) can be successfully applied for monitoring the status of the heart and blood vessels. They serve as an alternative to large-scale monitoring equipment, enabling the implementation of personalized medicine.

The triboelectric nanogenerator (TENG) used in medical devices is unique in its ability to utilize energy that would otherwise be wasted. It can harness energy generated by the contraction of arteries to sense abnormal heart rhythms and to power itself. The self-powered endocardial pressure sensor (SEPS) based on TENG, which is integrated with a surgical catheter for minimally invasive implantation, was presented by Zh. Liu [277], where nano-PTFE film with ultrathin gold (Au) layer (50 nm) was deposited on the back; then, it was treated by the inductively coupled plasma (ICP) method, and 3D ethylene-vinyl acetate (EVA) copolymer film (500 µm) was employed as the spacer layer. According to U.V. Romero’s [190] investigation, the stent-TENG operating mechanism can be divided into few steps: in the stent, when there is no arterial pressure and during vasodilation, no electrons are generated; with occurring arterial constriction (vasoconstriction), electrons are coming from PDMS to PVDF, and a negative charge is created on PVDF and a positive charge on PDMS. A potential difference is generated between the electrodes, and then the electrode layers are separated. An external circuit is used to allow the electrical current to flow. The potential obtains higher values, the current returns to zero, and TENG reaches the released condition. Macro, micro, and nano wrinkles have been fabricated to provide electrodes and active tribo-layers with high tensile ability for sensors based on PEDOT: PSS with rGO, MXene for TENGs (MW-STENGs), with stable MW-carbon-based electrodes/PDMS (MW-rGO/PDMS, MW-SWCNTs/PDMS, and MW-MXene/PDMS) to form the other tribo-electrode layer [128]. Miniaturized SEPS can reduce damage to the implanting tissues, resulting in a minimally invasive surgical method. With such a device, life-threatening arrhythmias can also be detected in time, based on sudden changes in endocardial pressure (EP). Implantable electronic devices have some disadvantages, including a limited battery life. A surgical procedure is performed periodically to replace the battery, causing patients to suffer and increasing the healthcare costs. A self-powered single-cell implantable triboelectric active sensor (iTEAS) that can continuously monitor a wide range of physiological parameters with efficient use of internal mechanical energy was presented by Y. Ma [278] for cardiac arrhythmias such as atrial fibrillation and premature ventricular contraction. The applied polymers for this sensor are Kapton PI, PDMS, and n-PTFE as the triboelectric layer, with metals such as Au, Ti, and Al. As with most implantable products, the biocompatibility of the device was tested in vivo after 2 weeks of implantation, demonstrating its suitability for practical use. Elastic fiber sensors have attracted interest due to their easy application in wearable sensors with human–machine interfaces and implantable devices. A fiber sensor proposed by Y. Zhang [77] achieved GF ≈ 1950, a broad strain-sensing range (400%), and high durability over 1000 stretching and bending cycles. The endocardial pressure has significant clinical implications for patients with heart failure. In vivo biomechanical energy harvesting is a solution for obtaining sustainable electrical energy to power implanted devices. The presented triboelectric nanogenerator was made using Kapton, PTFE, and PDMS with parylene encapsulation, as described by Q. Zheng [279]; this device can directly indicate the physiological heartbeat behavior. The conductive micro-nano carbon ink, printed on an elastomer, is fabricated as a force sensor proposed for use in smart gloves for human–machine interfaces and rehabilitation treatment. The product responds to different gestures, for example, Morse code by finger bending [280]. An indium tin oxide single electrode was applied as a pulse sensor, as proposed by X. Cui [281], working as a triboelectric nanogenerator, where incident waves and reflected waves are observed successfully.

## 5. Discussion and Conclusions

By evaluating the collected information and data from the discussed publications, the following conclusions can be stated for force sensors integrated into cardiovascular stents, as well as based on successful applications in surgery provided by the authors of the selected articles.

Sensors implanted in cardiovascular stents perform the following functions:

- Pulse registration,
- Blood pressure measurement,
- Tissue movement measurement,
- Micro-deformation detection,
- ECG and EMG data collection.

Improvements are needed for implantable cardiovascular stents with integrated force sensors, as follows:

- Implantable devices are a potential unwanted source of health status complications;
- Short-term postoperative monitoring requires temporary implantable devices that are subsequently removed from the body and pose additional risks.

The development of devices poses certain requirements, as follows:

- Biodegradable devices for short-term monitoring;
- Long-term devices with reliable long-term functioning;
- Supporting equipment for implantable health monitoring devices.

Polymers for implantable device application can be selected to improve the physical properties:

- Biocompatible polymers;
- A combination of polymers resulting in better application and device performance;
- A combination with nanostructures to improve device performance.

Hence, polymers have become an integral part of modern medical equipment. The polymer-based force sensors in cardiology are being developed in several directions:

- For personal self-monitoring systems, which can be implanted or integrated into wearable gadgets, with data collection and monitoring from the medical institution;
- systems utilizing AI and ML, which do not require exceptional accuracy, due to recommendation-type devices at the level of monitoring required by the health condition;
- Ultrasonic communication for data collection;
- Other types of sensors incorporated into cardiovascular stents or devices for healthcare personnel, dependent on health status.

The following solutions have been chosen for force sensor data collection and servicing:

1. Machine learning algorithms, AI, and IoT are essential tools being analyzed to support long-term cardiovascular care through real-time monitoring and database collection for further research;
2. The health status information collected from implanted devices with sensors, whose parameters can be read remotely.

The variety of solutions and new devices available promises greater flexibility in using equipment that is most suitable for a given patient. Hence, one of the currently relevant topics of sensor development is the reliability over the broadest possible operating range in various environmental conditions.

To assess which type of sensor is in higher demand or which kind of sensor needs improvement, studies should be conducted based on regional or traditional medicine requirements with specific needs for sensors:

- Implanted biodegradable sensors to monitor postoperative conditions in the short term;
- Long-term or lifelong stents with force sensors that maintain the quality of parameter recording throughout the entire period.

Three-dimensional printing technologies are increasingly being chosen for the production of force sensors integrated into stents, which require smaller production facilities and are more environmentally friendly.

Despite the wide variety of polymers suitable for contact with internal organs, most publications have used PDMS in combination with other polymers.

When discussing nanostructures used for sensors, two trends stand out. When a stent with a sensor is implanted for a long time, the aim is usually to use metallic nanostructures, and their toxicity is addressed by other technological solutions that protect against the toxic effects. When a sensor with a stent is implanted for a short time, in order to collect data on the success of invasive treatment procedures, materials that are harmless to the body are chosen, which naturally decompose and are removed from the body without additional surgical interventions.

Thus, the summary of the entire study is as follows.

With the successful application of hydrogels in sensors, the need arises for implanted biodegradable sensors to monitor postoperative conditions in the short term, which can be utilized naturally without ancillary surgery. As mentioned in some of the discussed publications, one of the main problems with all implantable devices is the potential for undesirable side effects. The quality of cardiovascular stents with integrated sensors, as well as becoming a safe “friendly” device for the human body, is still in the hands of developers. The reliability and quality of cardiovascular stents, as well as the diversity of patient needs, can be enhanced by using a combination of materials, both newly developed and established, in conjunction with nanomaterials, nanostructures, or emerging technologies. One of the most critical directions for increasing the diversity of polymer sensor compositions is the use of nanostructures, which can be achieved by incorporating additional materials and by forming nanostructures directly from the polymers themselves. Among the nanomaterials used in the production of polymer force sensors, there is a clear leader—carbon nanomaterials. Among nanostructures formed from the polymer itself, the most commonly referred to are nano wrinkles and nano threads. Mechano-nanomedicine is not addressed in this research, but it is impossible not to mention this promising field of polymer application in healthcare.

The diversity of materials, combined with the unique properties of polymers, enables a more reliable replacement of metal stents with sensors and opens up the possibility of stent resorption when its functions are no longer needed. This is especially useful when stents are used in the postoperative period, and the absence of a stent removal procedure reduces the risk to the patient’s health. Thus, polymers are an integral part of healthcare product manufacturing, the influence of which is becoming increasingly significant every year.

Further development of polymer-based sensors for heart and blood vessel monitoring can lead to the development of new designs of these sensors, which now are based on a metal-inspired form. The appearance of new polymers, like biopolymers, suitable for 3D printing leads to personalized medicine development and broader accessibility.

## Figures and Tables

**Table 1:** PDMS in healthcare sensors.

Polymer	Nanostructure	Measured Quantity and Sensor Properties	Ref.
PDMS	AgNWs)/PEDOT:PSS/PU	Film	[171]
PDMS	Ag@OH-f MWCNTs	Sandwich wrinkled, GF = 412.32	[172]
Pd-CNTs-rGO/PDMS sponge	rGO/CNTs	GF = 27.89	[173]
PDMS/PI	Au/Cr	WVTR = 486.17 g^−1^ d^−1^ m^−1^	[165]
PDMS	GNP	GF = 69	[160]
PDMS/rGO)/PEI/PI/Ag-plates	GO and rGO	Spray-dip process, bending-type	[174]
PDMS		Fiber optic sensor, to measure RPTT, BPTT, and the transit time DBRPTT	[175]
PDMS	Cu, Ni	Capacitive pressure; motion in gloves and shoes	[176]
PDMS	cellulose	Wide range force sensor	[177]
PDMS/CB	micropattern	e-skin type sensor	[178]
PDMS/UCNPs/SPOFs	UCNPs: NaYF4: Yb, Er, and a shell of NaYF4	Radiometric temperature sensing	[179]
PEDOT: PSS, PDMS, PVC	microfluidic channel	Force sensor, smart bandage for respiratory diseases, GF = 12	[180]
PDMS	rGO	Pulse, GF = 44.01	[181]
PDMS/PVA/SBMA fiber	nanofiber	Force, sensitivity −0.68 mV/%	[182]
AgNPs/silica xerogel film/PPy/CNT/PDMS,	CNT	Pressure sensor, sensitivity of 0.615 kPa in the range of 2–11 kPa,	[121]
PDMS/GaN	micronanostructured PDMS	Pressure sensor; sensitivity 94.4 μA/N in 0–1 N range; recovery time 2.8 ms	[183]
PDMS/parylene/iridium oxide	nanowrinkles	Pulse. ECG; sensitivity up to 51.03 mV/pH	[184]
PDMS, Ti alloy, Cu, PTFE	Au	Capacitive, pressure sensor, pulse, flow	[185]
PDMS, AorfixTM-stent	Au	IoT, impedance shift, aneurysm repair	[186]
PDMS micro-pyramids, Pi ink, parylene-C for Au layer formation	Au	Capacitive pressure, a resonant circuit to send a wireless signal, and blood flow	[187]

**Table 2:** Polymers in healthcare sensors.

Polymer	Nanostructure	Measured Quantity and Sensor Properties	Ref.
Co/Cr– Poly(ɛ-caprolactone) (PCL)–Co/Cr	Cr/Au layers	Pressure sensor for real-time blood pressure monitoring	[37]
PCL MEMS	CNT	Capacitive pressure for hemodynamic changes	[206]
Biodegradable polymer	Cr/Au	Capacitive pressure sensor for intravascular conditions and early conditions	[207]
PDA	CNT	Muscle movement sensing	[208]
PI/parylene/PDMS	--	Sponge, smart prosthesis	[209]
ISFET/PET	InGaZnO ∼30 nm + Al_2_O_3_ layer ∼50 nm	Non-invasive amorphous thin film	[210]
Pebax	MWCNT/Ag	Compression piezoresistive strain, motion recognition by synergic effect	[211]
PAAm/casein hydrogel	Li Cl	Movement and physiological signals	[212]
Au/CPI	Au ∼60–80 nm	Fabric capacitive wireless blood pressure sensor, fingertip pulse monitoring	[213]
GPANI-PVB-PET	ITO	Temperature and pressure detection	[214]
PU/PEDOT: PSS	Graphene	Fiber, temperature	[215]
PVDF	GO sheets	Ferroelectric, piezoelectric, pyroelectric, and piezoresistive sensing for static and dynamic mechanothermal signals	[216]
100-μ PVDF	300 nm layer Cu, Ti	Pressure	[217]
PCL	CNT	Pressure	[218]
PTFE/Parylene C	W	Cardiac function	[219]
PVDF nanofiber membranes, density (1.78 g/cm^3^)	β and γ phases	Piezo-Capacitive tactile Cu electrodes, membrane sandwiched in Kapton PI films	[220]
PVDF; PDA@BTO/PVDF	BaTiO_3_, BTO	Motion monitoring	[221]
PVDF/MXENEs/PET	MXENE nanofiber	Blood pressure	[222]
PEDOT: PSS/PVA/G	graphene	Optoelectronics for autonomous assistants	[223]
TPU	TPU nanofiber, AgNW	3D capacitive pressure sensor	[224]
TPU, Ag, microcracks	Nanopaper, nanocellulose, AgNW	Temperature sensor	[225]
Elastomer, HSPS, PDMS, FEP/Ag; PET/Ags	Electrostatic nanogenerator	Blood pressure, wearable	[226]
Elastomer	CNT	Pressure capacitive	[227]
PANI NFs	PANI NFs	Force and temperature sensor GF = 18.28	[202]
PEDOT: PSS, GOPS, CYTOP, AgNP	AgNP	Temperature	[228]
PPy/MWCNT/PU	MWCNT, AAO pilar;	Pressure, sensitivity 208.353 kPa^−1^	[229]
PP nanowoven/carbon ink	PPnanowoven	Temperature, pressure, sensitivity 0.228 kPa^−1^	[230]
PU	PU@AgNWs@SLG	GF = 0.47 to 2.39; sensitivity ∼0.009 kPa^−1^	[231]

**Table 3:** Polymers in biological parameter measuring sensors.

Polymer	Nanostructure	Declared Sensor Properties	Ref.
Silicone rubber/with PDMS, MWCNTs	MWCNTs	Piezoresistive, compression	[249]
Cotton	SWCNT, MACNTs	resistive	[250]
SF@MXene	Ti_3_C_2_T_x_	3D cross-linked for small deformations	[251]
PAA, SF, MXene	MXene	Strain sensor, GF = 6.04	[252]
Cellulose/GO	GO	Multifunctional	[253]
SCMC/PAA	MXene, Ti_3_C_2_T_X_	GF = 5.79 to 40.36	[254]
SA/GE sponge/PVDF (SGSP)	CIPs	GFcom = −0.76 ± 0.05 and −1.25 ± 0.16	[255]
Silk/PEDOT	CeO2/silk fiber	GFp1 = −2.861% kPa^−1^; GFp2 = −0.845% kPa^−1^,	[256]
Silk/DES	Nanofibers	Blood pressure, sensitivity 138.5 kPa^−1^	[257]
SA/Pam/CuNPs	CuNPS	Tensile strength of 0.42 MPa, electrical conductivity of 2.4 S m^−1^	[258]

**Table 4:** Combinations of natural and synthetic polymers suitable for healthcare sensors.

Polymer	Application	Sensor Properties	Ref.
ASDP/PVA/PHMG	pulse	Pulse, ECG	[261]
Starch, PVA, AlCl_3_, [Emim]Ac	pulse	GF = 5.93	[262]
Starch/PVA/borax, or SPB	force, movement	GF = 1.02 at 110–200% strains	[263]
Cellulose, gelatin	blood pressure	HG-TENG, power density of 57.8 µW/cm^2^	[76]
Gelatin/OCMC	movement	GF = 0.18 to 0.86	[234]
MC/TA@CNCs	movement, force	GF = 1.63	[235]
SF/GMA	ECG, EMG, motion	Strain 414.6%	[264]
Silk sericin protein, F-SS/ZnO/PVA-based piezoelectric films	ECG	Peak power density of 218.5 µW/m^2^	[265]
Candelilla wax (C_w_), beeswax (B_w_), MoO_3_	micro-deformation	GF ≈ 100	[86]

## Abbreviations

The following abbreviations are used in this manuscript:

AA	Acrylic acid
AAO	Alumina template
ADSP	Acetylated di-starch phosphate
AI	Artificial intelligence
AlN	Aluminum nitride
Ag@OH-f MWCNTs	Nanosilver (Ag)-coated hydroxyl-functionalized multi-walled carbon nanotubes
AgNWs	Silver nanowires
BCMC	Bias-free cardiac monitoring capsule
BPTT	Brachial artery transit time
CB	Carbon black
CIPs	Carbonyl iron particles
CNDs	Carbon nanodots
CPI	Colorless polyimide
CuNPs	Copper metal nanoparticles
CYTOP	Fluorinated polymer passivation
CVD	Cardiovascular diseases
DES	Ionic deep eutectic solvents
DBRPTT	Transit time difference between the radial and the brachial artery
EB	Elastic band
ECG	Electrocardiogram
EMG	Electromyography
[Emim]Ac	1-ethyl-3-methyl imidazolium acetate
FEP	Fluorinated ethylene propylene
FSS	Functionalized silk sericin
GF	Gauge factor
G	Graphene
GMA	Glycidyl methacrylate
GNPs	Graphene nanoplatelets
GO	Graphene oxide
GPANI/PVB	Polyaniline-graphene/polyvinyl butyral film
GOPS	(3-glycidyl oxy propyl) trimethoxysilane
HG-TENG	Hydroxyethyl cellulose (HEC) and gelatin-based TENG
HF	Heart failure
HDL	High-density lipoprotein
HPFS	Hydrogel-based polymer fiber strain
HSPS	Hierarchical self-powered pressure sensor
ISFET	Ion-sensitive field-effect transistor
ITO	Indium tin oxide
IVC	Inferior vena cava
Kapton (PI)	Kapton poliimide
LBG	Locust bean gum
MC	Methylcellulose
MW-STENGs	Macro-micro-wrinkled stretchable TENGs
MEMS	Micro-electromechanical systems
MWCNTs	Multi-walled carbon nanotubes
PTFE	Polytetrafluoroethylene
PPy/MWCNT/PU	Polypyrrole/multi-walled carbon nanotube/polyurethane
NHMS	Nitinol health monitor sensor
NFs	Nanofibers
NPs	Metal oxide nanoparticles
OCMC	Oxidized sodium carboxymethyl cellulose
PAAm	Poly(acryl amide)
PANI	Polyaniline
PCL	Poly(ɛ-caprolactone)
PDA	Polydopamine
[P(DPP2ODT2-T)]	Poly{3-([2,2′:5′,2″-terthiophen]-5-yl)-2,5-bis(2-octyldodecyl)-2,5-dihydropyrrolo [3,4-*c*]pyrrole-1,4-dione-6,5″-diyl}
PDMS	Polydimethylsiloxane
PEDOT: PSS	Poly(3,4-ethylenedioxythiophene): poly (styrene-sulfonate)
PMMA	Poly (methyl methacrylate)
Pebax	Poly-ether-*b*-amide
PEI	Polyethyleneimine
Pebax	Poly(ether-*b*-amide)
PEN	Polyethylenenaphthalene
PET	Polyethylene terephthalate
PI	Polyimide
PHMG	Poly(hexamethylene biguanide) hydrochloride
PLA	Polylactic acid
PP	Polypropylene
PU	Polyurethane
PVA	Poly(vinyl alcohol)
PVDF	Poly(vinylidene fluoride)
PVC	Polyvinyl chloride
rGO	Reduced graphene oxide
RPTT	Radial artery transit time
SA	Sodium alginate
SGS	Sodium alginate (SA)/gelatin (GE) sponge
SLG	Single layer graphene
SMS	Single-mode–multi-mode–single-mode
SCMC	Sodium carboxymethyl cellulose
SPB	Starch/PVA/borax
SPOFs	Stretchable polymer-based optical fibers
SBMA	2-(methacryloyloxy)ethyl]dimethyl-(3-sulfopropyl) ammonium hydroxide
SF	Silk fibroin
SWCNT	Single-walled carbon nanotubes
SF@MXene	Silk fibroin@Ti_3_C_2_T_x_ MXene
TA@CNCs	Tannic acid-coated cellulose nanocrystal
TPU	Thermoplastic polyurethane
UCNPs	Thermal-sensitive upconverted nanoparticles
WVTR	Water vapor transmission rate
